# Exemestane-Everolimus-Induced Angioedema in a Woman With Metastatic Breast Cancer: A Case Report and Review

**DOI:** 10.7759/cureus.48628

**Published:** 2023-11-10

**Authors:** Yisroel Y Grabie, Adham Ahmed, Sudeep Acharya, Matthew H Flamenbaum

**Affiliations:** 1 Department of Internal Medicine, Staten Island University Hospital, Northwell Health, Staten Island, USA; 2 Department of Internal Medicine/Pulmonary and Critical Care, Donald and Barbara Zucker School of Medicine at Hofstra/Northwell, Staten Island, USA; 3 Department of Pulmonology/Critical Care, Donald and Barbara Zucker School of Medicine at Hofstra/Northwell, Staten Island, USA

**Keywords:** mechanical ventilation, everolimus, severe respiratory distress syndrome, airway intubation, drug-induced angioedema

## Abstract

Dual exemestane-everolimus therapy has been shown to confer a progression-free survival benefit in women with refractory advanced hormone-receptor-positive breast cancer. Patients with breast cancer may experience several other comorbidities, including hypertension, for which angiotensin-converting enzyme inhibitors (ACE-I) are a first-line therapy for hypertension of cardiovascular and/or renal etiology. One rare but feared side effect of ACE-I is severe angioedema due to decreased bradykinin degradation, which can lead to respiratory collapse. Several single-center case series have previously suggested that the use of everolimus in conjunction with ACE-I may lower the threshold for angioedema development. We report our experiences managing a 71-year-old with metastatic breast carcinoma and hypertension who presented with severe angioedema after the combined use of exemestane-everolimus with lisinopril.

## Introduction

Exemestane is a novel aromatase inhibitor that acts by blocking the aromatization of androstenedione to estrone in tissues. The drug is highly specific and has been previously reported to block in vivo aromatase conversion by a mean rate of over 97% [1]. When given to postmenopausal women with estrogen-receptor-positive breast cancer, switching to exemestane after two to three years of tamoxifen has been shown to significantly reduce the risk of local or metastatic recurrence, the development of contralateral breast cancer, and death compared to the standard five-years of tamoxifen therapy [2]. Conversely, everolimus is a rapamycin analog that functions by binding cyclophilin FKBP12 to form a complex with serine-threonine kinases and mTOR (mammalian target of rapamycin) to inhibit downstream signaling [3]. While currently approved as an antitumor agent in many solid organ malignancies, the landmark BOLERO-2 trial demonstrated its strong synergistic effect when given together with exemestane for nonsteroidal aromatase inhibitor-resistant advanced hormone-receptor-positive breast cancer [4]. In that study, patients treated with dual exemestane-everolimus therapy experienced a 6.9-month median progression-free survival compared to 2.8 months in the control group.

Angiotensin-converting enzyme inhibitors (ACE-I) are a hallmark therapy in patients with cardiovascular and renal disorders and a first-line therapy for hypertension with concurrent diabetes mellitus. These agents lower mean arterial blood pressure by inhibiting the conversion of angiotensin I to angiotensin II in the lung, decreasing the vasoconstrictive effect of angiotensin II and blocking downstream aldosterone effects [5]. Despite their therapeutic efficacy, ACE-I may cause several side effects, including oropharynx and upper respiratory tract angioedema in susceptible populations. The mechanism by which this occurs is believed to be caused by the blockage of bradykinin degradation by angiotensin-converting enzyme [6]. The angioedema caused by bradykinin buildup is notable for its absence of flushing, bronchospasm, and urticaria, which may distinguish it clinically from mast-cell-induced angioedema. Interestingly, there is also some center-level data suggesting that the combined use of ACE-I in patients receiving mTOR inhibitor therapy may predispose patients to angioedema, although the underlying pathomechanism is not fully understood [7]. Herein, we report the management of a 71-year-old female with metastatic breast cancer who developed acute angioedema following the simultaneous use of exemestane-everolimus and lisinopril.

## Case presentation

**Figure 1:**
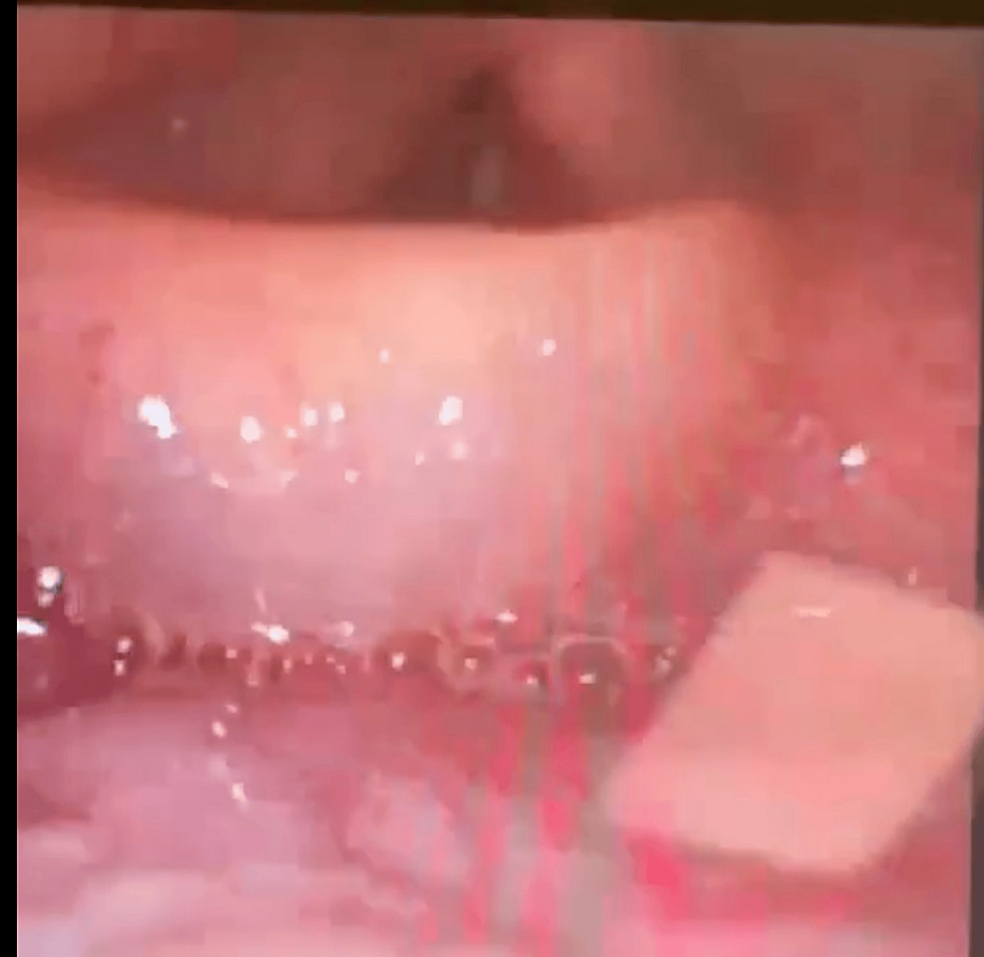
Edematous epiglottis and glottis during endotracheal intubation.

A 71-year-old female with a past medical history of metastatic breast carcinoma (on exemestane-everolimus dual therapy), hypertension (on lisinopril), dyslipidemia, and cerebrovascular disease presented acutely to our emergency department with tongue and laryngeal edema with decreased vocalization. History was provided by her husband at bedside who stated that she awoke at 5 am with dysphagia and a sensation of swelling around her throat. She took Benadryl and went back to sleep but woke up a few hours later with worsening swelling. The patient’s last dose of lisinopril was the previous evening, but he confirmed that she had been on the dose for years without incident. The patient was nonverbal during the initial encounter but was responsive and communicated by writing. She endorsed drooling and dyspnea with the angioedema. The patient was hemodynamically stable and saturating 99% on room air. Due to the risk of complete airway obstruction, the patient was intubated. Figure 1** **displays the patient's edematous epiglottis and glottis, which were visible during intubation, and placed on mechanical ventilation; a chest X-ray was used to confirm successful placement. Labs were all within normal limits. The patient was given dexamethasone in the ED and admitted to the intensive care unit (ICU).

In the ICU, the patient was started on Solu-Medrol 60 mg with Benadryl twice a day. The leading differential at the time was idiosyncratic angioedema secondary to angiotensin-inhibiting enzyme therapy. Interestingly, her C1 inhibitor and tryptase levels came back within normal limits. A comprehensive review of her medication showed that she was currently on 25 mg extended-release metoprolol, 25 mg hydrochlorothiazide, 20 mg lisinopril, 10 mg everolimus, and 25 mg exemestane. The patient had taken these drugs (including the three anti-hypertensive agents, metoprolol, hydrochlorothiazide, and lisinopril) at controlled doses concurrently for a few years without any side effects, acute hypotension, or other adverse cross-interactions. The patient’s husband reported her breast cancer therapy had been modified approximately two months prior but denied any other changes in medications. The otorhinolaryngology service was consulted for and cleared the patient to be extubated with discontinuation of her lisinopril.

## Discussion

Carcinoma of the breast is one of the most common types of cancer by prevalence and is currently the leading cause of cancer mortality in females [8]. While the metastatic spread is a poor prognostic indicator, there has been an improvement in survival and recurrence rates in these patients with the advent of effective adjuvant therapies, including but not limited to selective aromatase inhibitors [9], direct estrogen blockers [10], and mTOR inhibitors [11]. The combined use of exemestane-everolimus for advanced hormone-receptor-positive breast cancer has been shown to significantly improve patient progression-free survival. This synergistic benefit may be due to the differing mechanisms working in conjunction to reduce malignant proliferation; while exemestane functions to reduce the conversion of androgens into estrogens, everolimus works to inhibit downstream intracellular signaling pathways that are upregulated in breast cancers. Notably in the BOLERO-2 trial, researchers did observe an elevated rate of adverse effects with dual therapy compared to exemestane alone, including stomatitis, cough, hyperglycemia, and diarrhea [4].

We report our experience managing a 71-year-old female who presented acutely with tongue and upper airway swelling associated with an inability to talk. The patient’s past medical history was significant for hypertension managed with lisinopril for years and metastatic breast carcinoma, for which the patient was currently on exemestane-everolimus therapy. At the bedside, the patient’s husband recalled that her breast cancer regimen was recently modified but both he and the patient did not know further details. The patient was given steroids and intubated due to the risk of respiratory compromise. She responded well and was extubated after one day. C1-esterase inhibitor deficiency was ruled out, but given the lack of information regarding her exemestane-everolimus dosage, the patient was advised to switch her lisinopril to amlodipine in the meantime and follow up outpatient to adjust her adjuvant breast cancer regimen.

While several reports have previously shown an elevated risk in patients taking tamoxifen [12,13], literature on exemestane-everolimus-induced angioedema is limited. Previously in 2005, Fuchs and colleagues documented six cases of lingual angioedema in patients receiving everolimus for post-heart transplant immunosuppression [14]. In all cases, the symptoms occurred within two months and were associated with bullae development along the lateral tongue and petechial bleeding. Everolimus had to be discontinued in only one patient, with the other five experiencing symptom resolution with supportive therapy. Interestingly, the authors reported that two patients were on an ACE-I (enalapril 10 mg/ramipril 5 mg) and one patient was on an angiotensin-II receptor blocker (losartan 50 mg). More recently, a 2015 case report by Dievel et al. reported the management of an 89-year-old male with metastatic breast cancer who developed unilateral angioedema of the tongue after the combined use of exemestane-everolimus with lisinopril [15]. In that report, the patient was conservatively managed with a one-time dose of IV methylprednisolone, antihistamines, and switching of his lisinopril to an angiotensin-II receptor blocker. In our case, the patient presented with much more severe angioedema, involving the tongue and throat, necessitating intubation. Interestingly, our patient was on her ACE-I for several years without ever experiencing an episode like this. Our theory is that the combined use of exemestane-everolimus lowered her threshold for angioedema, although this needs further investigation. Of note, prior studies have shown an increased risk for angioedema in patients taking combined mTOR inhibitors with ACE-I [7].

## Conclusions

To conclude, we report a unique case of acute angioedema in an elderly lady with metastatic breast carcinoma and hypertension after the simultaneous use of exemestane-everolimus and ACE-I. As the use of adjuvant exemestane-everolimus therapy for advanced breast cancer increases due to its efficacy, it is crucial for in-hospital services to swiftly and accurately diagnose any dangerous adverse effects. Moreover, integration of these agents into existing medical regimens, such as ACE-I, warrants careful consideration and multidisciplinary collaboration between primary care providers, cardiologists, and hematologist-oncologists. While we hope this case helps provide further insight for clinicians, further investigation is needed to ascertain the underlying pathophysiology, prevention, and management of this acute idiosyncratic event.
